# Hypothalamic control of energy expenditure and thermogenesis

**DOI:** 10.1038/s12276-022-00741-z

**Published:** 2022-03-17

**Authors:** Le Trung Tran, Sohee Park, Seul Ki Kim, Jin Sun Lee, Ki Woo Kim, Obin Kwon

**Affiliations:** 1 Departments of Oral Biology and Applied Biological Science, BK21 Four, Yonsei University College of Dentistry, Seoul, 03722 Korea; 2 Department of Biomedical Sciences, Seoul National University College of Medicine, Seoul, 03080 Korea; 3 Departments of Biochemistry and Molecular Biology, Seoul National University College of Medicine, Seoul, 03080 Korea

**Keywords:** Molecular neuroscience, Endocrine system and metabolic diseases

## Abstract

Energy expenditure and energy intake need to be balanced to maintain proper energy homeostasis. Energy homeostasis is tightly regulated by the central nervous system, and the hypothalamus is the primary center for the regulation of energy balance. The hypothalamus exerts its effect through both humoral and neuronal mechanisms, and each hypothalamic area has a distinct role in the regulation of energy expenditure. Recent studies have advanced the understanding of the molecular regulation of energy expenditure and thermogenesis in the hypothalamus with targeted manipulation techniques of the mouse genome and neuronal function. In this review, we elucidate recent progress in understanding the mechanism of how the hypothalamus affects basal metabolism, modulates physical activity, and adapts to environmental temperature and food intake changes.

## Hypothalamus: Control of energy expenditure

The hypothalamus is a key regulator of metabolism, controlling resting metabolism, activity levels, and responses to external temperature and food intake. The balance between energy intake and expenditure must be tightly controlled, with imbalances resulting in metabolic disorders such as obesity or diabetes. Obin Kwon at Seoul National University College of Medicine and Ki Woo Kim at Yonsei University College of Dentistry, Seoul, both in South Korea, and coworkers reviewed how metabolism is regulated by the hypothalamus, a small hormone-producing brain region. They report that hormonal and neuronal signals from the hypothalamus influence the ratio of lean to fatty tissue, gender-based differences in metabolism, activity levels, and weight gain in response to food intake. They note that further studies to untangle cause-and-effect relationships and other genetic factors will improve our understanding of metabolic regulation.

## Introduction

Homeostasis is the steady state of conditions for the optimal function of an organism, including humans. This concept appears to be a static state, but it is a dynamic equilibrium actively regulated by elaborate systems with complex feedback mechanisms. Energy homeostasis is one of the balances that should be maintained within a narrow range in the body. Energy imbalances result in metabolic diseases such as obesity and diabetes mellitus.

Energy homeostasis is achieved by balancing energy expenditure and energy intake. The hypothalamus is a brain region thought to play a critical role in the regulation of energy homeostasis^1^. In this review, we summarized the components of energy expenditure at the organism level and how they are controlled by the hypothalamus. The hypothalamic regulation of homeostatic and hedonic feeding will be covered by another review paper in this Special Feature Series by Ahn et al.

## Components of energy expenditure

Total energy expenditure comprises resting metabolic rate, the thermic effect of physical activity, and adaptive thermogenesis. The resting metabolic rate (RMR) is the minimal energy expenditure for living cells and tissues working in a resting state. The thermic effect of physical activity means the energy expenditure and heat production during physical activities, even if the activity is related to only a change in posture or fidgeting^2^. Adaptive thermogenesis is regulated thermogenesis in response to environmental changes such as a caloric surplus or cold temperatures. Table 1 summarizes the various components of total energy expenditure and their relative proportions. In another division, energy expenditure can be divided into obligatory and facultative thermogenesis: the former refers to the mandatory response for daily body function, whereas the latter refers to the additional responses beyond obligatory thermogenesis and is related to the adaptive increase in energy expenditure^3^.

**Table 1:** Components of energy expenditure.

% approx.	Component	Subcomponent	Definition	Major site	Related hypothalamic nucleus	Category
70	Resting metabolic rate	Standard metabolic rate	The amount of energy at rest in a thermoneutral environment	Skeletal muscle	PVN, ARC, VMH	Obligatory
		Thermic effect of food	The heat generated during the digestion, absorption, processing of food	Gastrointestinal tract		
20	Energy expenditure for physical activity	Nonexercise activity thermogenesis (NEAT)	The heat generated by spontaneous physical activity (SPA)	Skeletal muscle	LH, PVN, ARC, VMH	Obligatory or Facultative
		Exercise activity thermogenesis	The heat generated by exercise			Facultative
10	Diet-induced thermogenesis	–	The heat produced in response to excess caloric intake	BAT	ARC, VMH, POA, DMH	Facultative
Variable	Cold-induced thermogenesis	Shivering thermogenesis (ST)	The heat for protecting the organism from cold exposure by shivering	Skeletal muscle	POA, DMH, VMH, PVN	Facultative
		Nonshivering thermogenesis (NST)	The heat generated to adapt to cold	BAT		Obligatory or Facultative

*ARC* arcuate nucleus of the hypothalamus, *BAT* brown adipose tissue, *DMH* dorsomedial hypothalamus, *LH* lateral hypothalamus, *POA* preoptic area, *PVN* paraventricular hypothalamus, *VMH* ventromedial hypothalamus.

### Resting metabolic rate

Even while we are resting, we need energy to stay alive. The largest component of energy expenditure is the RMR, which accounts for ~70% of the total energy expenditure. The RMR is the amount of energy per unit time that an organism needs to keep the body functioning at thermoneutrality during food digestion^4,5^. The RMR is highly and positively correlated with lean mass^6–9^. Other body compositions, such as fat mass, height, sex, age, and hormonal factors, can affect the RMR^7,10,11^. The standard metabolic rate (SMR) is similar to the RMR, but the organism is in a fasted state for at least 12 h^4^, and the SMR demonstrates the minimum energetic cost of living^12^. The thermic effect of food is a type of obligatory thermogenesis and results from the digestion, absorption, and storage of ingested nutrients after a single meal. The RMR can be calculated as the sum of the SMR and the thermic effect of food.

### Physical activity

The thermic effect of physical activity accounts for 10–20% of the total daily energy expenditure^13^. In humans, at 1.5–2 h after physical activity, metabolism can increase 10–20% compared to its level before exercise^14^. Thermogenesis from physical activity includes nonexercise activity thermogenesis (NEAT) and exercise-induced thermogenesis. Spontaneous physical activity (SPA), such as fidgeting and maintaining or changing posture, can induce NEAT^15^.

### Adaptive thermogenesis

This includes diet-induced thermogenesis (DIT) and cold-induced thermogenesis (CIT). In rodents, brown adipose tissue (BAT) is the major site for adaptive thermogenesis. Brown and beige adipose tissues have been defined and characterized extensively in both humans^16^ and rodents^17^ as thermogenic organs. Thermogenic adipocytes highly express uncoupling protein 1 (UCP1), which plays an important role in heat generation, especially in canonical adaptive thermogenesis. UCP1 localizes to the inner membrane of mitochondria and generates heat by dissipating the proton gradient from mitochondrial respiration (the ‘uncoupling’ reaction). UCP1 is vital, but not indispensable, for the maintenance of energy expenditure^18^. DIT is facultative thermogenesis beyond the thermic effect of food in response to excessive food intake, which might be a substantial part of the adaptive increase in energy expenditure^3^. CIT is heat generation in response to cold exposure to protect the organism itself. The acute response to cold is shivering, which means involuntary activation of skeletal muscle movement, but nonshivering thermogenesis (NST) becomes the main response after adaptation. This NST is further categorized into facultative NST and obligatory NST. The former term means a short-term increase in heat production because of cold exposure by activating BAT thermogenesis. On the other hand, the latter term means that the increase in body temperature is closely related to the basal metabolism of the organism, not an acute response to environmental temperature changes^19–21^.

## Hypothalamic control of basal metabolism

As mentioned above, basal metabolism accounts for the largest proportion of total energy expenditure. Approximately 70% of the respiration rate in the basal state is mitochondrial ATP production, ~20% is a mitochondria process to counteract mitochondrial proton leakage, and ~10% is a nonmitochondrial process^22^. Various factors are found to affect and determine resting energy expenditure, including body mass, age, sex, and the levels of several hormones, which have been primarily documented in human studies^7,10,11^. The hypothalamus exerts its effect through humoral and neuronal mechanisms, and the sections below illustrate the relevant hypothalamic regulatory mechanisms of basal metabolism.

### Lean mass and fat mass

Lean mass (i.e., fat-free mass, including muscle mass) is a leading determinant of basal metabolism in humans^6–9^. The ratio of lean mass to whole body weight is 60–70% in women and 70–80% in men, explaining why heat from skeletal muscle is the largest part of whole-body energy expenditure. Skeletal muscle metabolism determines not only basal metabolism but also adaptive thermogenesis, such as CIT, which will be discussed later in this review.

Likewise, in rodents, the basal metabolic rate (BMR) is more dependent on lean mass than fat mass, even in mice fed a high-fat diet^23^. Skeletal muscle can be increased by androgens, regulated by the hypothalamic–pituitary–gonadal axis. Hypothalamic gonadotropin-releasing hormone (GnRH) stimulates anterior pituitary gland secretion of luteinizing hormone, which stimulates the synthesis and secretion of testosterone in the gonads. The androgen receptor in the hypothalamus positively modulates fat-free mass in mice^24^, and androgen receptor-null male mice have suppressed energy expenditure, which results in late-onset obesity^25^. The hypothalamus expresses erythropoietin, which decreases with aging and dietary obesity. Central administration of erythropoietin increases lean mass and muscle function, while body weight and fat mass decrease^26^.

Fat mass is another major determinant of the metabolic rate in mice: obese organisms have higher basal metabolism than lean organisms. A previous paper showed that the contribution of fat mass to energy expenditure is absent in leptin-deficient ob/ob mice, which can be reversed by physiological leptin replacement. This result suggests that the contribution of fat mass to energy expenditure is leptin-dependent^27^.

Despite the importance of skeletal muscle and fat tissue on basal metabolism, more direct evidence is needed as to whether the hypothalamus regulates basal metabolism by determining lean and fat mass. Considering the nature of experimental studies, most of the data sampling of animal studies is cross-sectional (sampling at once), not longitudinal, and it is challenging to discover the exact causality between energy expenditure and lean mass and/or fat. For example, decreased energy expenditure with increased fat mass is simultaneously observed in a mouse model with genetic and/or pharmacological manipulation in the hypothalamus^28–30^. It is plausible to interpret energy expenditure as the cause (or mechanism) and decreased fat mass as the result, as fat mass is usually considered an ‘effect’, not a ‘cause’, of energy metabolism. In this manner, however, we cannot delineate whether and how body mass alterations by the hypothalamus affect basal energy metabolism. Therefore, more reliable experimental methods are needed to overcome this limitation.

### Height

Resting energy expenditure in human adults is typically predicted by other additional covariates, including height. This can be found even in one of the old equations for predicting resting energy expenditure (in kcal/day) as 13.8 × body weight (kg) + 5.0 × height (cm) − 6.8 × age (yrs) + 66.5 for men^10^. Therefore, generally, taller subjects have a larger fat-free mass than subjects with short stature. The growth hormone (GH)-insulin-like growth factor 1 (IGF-1) axis is the dominant endocrine system controlling linear growth during childhood^31^ and muscle mass^32^. The production and secretion of growth hormone from the anterior pituitary gland are under the control of hypothalamic GH releasing hormone (GHRH, positively) and somatostatin (negatively). Indeed, imaging studies of the hypothalamus (and pituitary gland) are required for the detection of anatomical defects in patients diagnosed with growth hormone deficiency^33^. An example of hypothalamic dysfunction with inadequate GH secretion is the short stature associated with thalassemia, which may result from iron deposits in hypothalamic neurons^34^.

Sox21 is an essential transcriptional regulator of the developing hypothalamus. A loss of Sox21 in mice leads to postnatal growth reduction with increased energy expenditure with normal physical activity and food intake^35^. This growth reduction may be nonendocrine given that all the other hypothalamic–pituitary axis were functionally intact^35^. Recently, Lee et al. showed that distal-less homeobox-1 (Dlx1) and its homolog Dlx2, transcription factors highly expressed in the mediobasal hypothalamus, are required for the specification of GHRH neurons^36^. Conditional Dlx1/2-null mice show a loss of GHRH neurons with higher somatostatin expression, smaller body size and lean mass, and lower energy expenditure with normal food intake^36^. Another study showed that insulin knockdown in the paraventricular nucleus of hypothalamus (PVN) of young mice suppresses growth with lower serum GH without changes in food intake, suggesting that parvocellular neurosecretory insulin neurons in the PVN have a crucial role in the regulation of GH production and body length^37^. These results all imply the importance of hypothalamic hormonal regulation of both body length and energy expenditure. Opposite results exist for positive or negative correlations between height and energy expenditure, as above. This phenomenon might be due to different clinical or experimental contexts in various studies, and more evidence is needed to delineate the causal relationship between height and energy expenditure under the control of the hypothalamus.

### Aging

Initial cross-sectional human studies described dramatic declines in the BMR with aging^11,38^, although later studies addressed that the degree of those declines was smaller than previously expected^39,40^. The loss of various lean tissues, including muscle and brain tissue, is related to the reduction in the BMR^40^. However, decreased lean mass cannot fully explain the lower basal metabolism in older ages, implying that aging per se may be associated with an alteration in tissue energy metabolism^39^.

The hypothalamus mutually influences aging. In the hypothalamus of aged mice, the expression of genes involved in reactive oxygen species (ROS) production and protein degradation is upregulated, while the expression of genes for synaptic function and integrity is downregulated^41^. On the other hand, an age-associated alteration in energy homeostasis and hormone balance can be derived from functional changes in specific groups of hypothalamic neurons^42,43^. In aged mice, proopiomelanocortin (POMC) neuronal activity is significantly reduced^44^, while age-dependent metabolic dysfunction can be mitigated by the rescue of the POMC gene in the arcuate nucleus (ARC)^45^. This finding represents a possible link between the hypothalamus and decreased energy expenditure with aging, considering that POMC-knockout mice show a BMR that is decreased by ~25%^46^.

Hypothalamic area genetic modulation studies have accumulated more evidence that the hypothalamus can serve as a target for the restoration of decreased energy expenditure with aging. The transfer of the brain-derived neurotrophic factor (BDNF) gene into the ARC and ventromedial nucleus of the hypothalamus (VMH) increases oxygen consumption even with lower physical activity, which indicates an elevated RMR﻿^47^. Viral expression of the glial cell line-derived neurotrophic factor (GDNF) gene in the hypothalamus of aged rats also increased energy expenditure despite reduced food intake^48^. Only a few studies have aimed to determine the effects of hypothalamic aging on energy expenditure, and more specific studies from this perspective are needed.

### Sex differences

When studying mechanisms that affect energy expenditure, males and females do not always have the same phenotype. These sex differences occur in estrogen signaling research in the hypothalamus and various hypothalamic signaling pathways^49–51^. Estrogen receptor alpha (ERα) signaling in the VMH is involved in energy homeostasis by regulating thermogenesis^49^. A recent study revealed that ERα is largely expressed in VMH neuronal populations that have sex-biased expression of reprimo (Rprm), a TP53- and ERα-regulated gene. Rprm expression can regulate core temperature in a sex-specific manner: female mice, but not male mice, with Rprm-siRNA injected into the VMH show an increased body temperature^51^. Phosphatidylinositol 3-kinase (PI3K) could mediate ERα signaling^52,53^. PI3K catalytic subunit deletion in steroidogenic factor 1 (SF-1) neurons causes obesity in female mice but not in male mice^50^. These female mice have decreased energy expenditure in the light phase without changes in food intake or locomotor activity in the same phase^50^. The acute effect of estrogen in SF-1-specific PI3K catalytic subunit KO female mice impairs the increase in total energy expenditure, while food intake or locomotor activity were not different between KO and WT mice^50^.

Sex differences in other signaling pathways that are not directly relevant to estrogen have also been reported. The 5-hydroxytryptamine 2c receptor (5-HT_2C_R) in ARC POMC neurons also regulates energy expenditure in a sex-dependent manner^30^. POMC deletion in the hypothalamus but the restoration of its expression in 5-HT_2C_R-positive neurons restores energy expenditure in male mice. On the other hand, female mice still have impaired total energy expenditure and resting energy expenditure similar to POMC deletion in whole hypothalamus^30^. G protein-coupled receptor 17 (Gpr17), suggested to be one of the transcriptional targets of forkhead box protein O1 (FoxO1) in the central nervous system^54^, shows different effects between sexes in POMC neurons^55^. POMC-specific Gpr17-KO female mice, but not male mice, fed a high-fat diet tend to increase energy expenditure in the light cycle^55^. This effect was reported without additional changes in food intake or activity in the light cycle^55^.

These various reports showed that sex-dimorphic basal metabolic regulation was under hypothalamic control, even though some studies suggested that sex is not relevant to BMR variations^7^. More detailed evidence about sex-dimorphic hypothalamic signaling may reveal differences in basal metabolism between sexes, and it is essential for researchers to keep in mind the potential differences between sexes when designing their studies.

### Thyroid hormone

Thyroid hormone [triiodothyronine (T_3_, biologically active form) and thyroxine (T_4_)] contributes to both obligatory and facultative thermogenesis^56^. In terms of resting energy expenditure, thyroid hormone stimulates the transcription of UCP genes directly, acting via thyroid hormone receptor binding sites, resulting in increased proton gradient leaks in mitochondria and thus the generation of heat^57^. This mechanism helps maintain body temperature and constitutes a significant part of the BMR. Thyroid hormone production is controlled by thyrotropin-releasing hormone (TRH), which is generated in the PVN^58^. TRH, which is released through the hypophyseal portal system, activates thyrotrophs in the anterior pituitary gland to release thyroid-stimulating hormone (TSH), which then stimulates thyroid hormone production in the thyroid gland (i.e., the hypothalamic–pituitary–thyroid (HPT) axis). Thyroid hormone receptors are expressed in TRH neurons in the PVN^59^, and changes in peripheral thyroid hormone levels provide feedback to the PVN to initiate compensatory regulation of TRH synthesis to maintain homeostasis^60^. When T_3_ stimulates the VMH by inhibiting 5′ adenosine monophosphate-activated protein kinase (AMPK), it was found that sympathetic nerves activate UCP1 in the mitochondria of brown adipose tissue, resulting in decreased body weight without changes in food intake^61^. This finding added another mechanism of thyroid thermogenesis, which had previously been known to be mediated mainly by muscular mitochondria and smooth endoplasmic reticulum Ca^2+^ ATPase (SERCA) in the endoplasmic reticulum^62^.

Tanycytes are a special type of glial cell lining the median eminence of the hypothalamus to control the crossing of intravascular substances into the brain^63^. Type 2 deiodinase catalyzes the transformation of T_4_ to T_3_, and this enzyme in tanycytes may be necessary for negative feedback to the HPT axis^64^. Tanycytes in the floor of the third ventricle (β2-tanycytes) express pyroglutamyl-peptidase II (TRH-degrading ectoenzyme), which is upregulated by thyroid hormone, providing negative feedback to the HPT axis^65,66^. The mechanism of tanycytes controlling TRH flux into portal capillaries is illustrated in another paper^67^. Therefore, tanycytes may modulate the effects of thyroid hormone on thermogenesis, which is significant for thermoregulation in hibernating mammals^68^. Other roles of hypothalamic thyroid hormone in energy balance regulation have been previously reviewed^69^.

When we interpret the outcome of energy expenditure in rodent studies, there is a critical point to be considered. Basal metabolism can be calculated under a thermoneutral environment, at ~30 °C for rodents^70–73^ and ~28 °C for naked humans^74,75^. However, most of the studies have been performed at room temperature (20–22 °C). Studies that measured basal metabolism in the thermoneutral zone to determine the exact resting energy expenditure have found room temperature to be a cold environment for rodents^29^. This difference could lead to ambiguous or different conclusions, and similar examples are found in mouse studies in which thermogenesis is genetically manipulated^76,77^. An adequate method of considering thermoneutral conditions while translating the findings in mice studies to human metabolic diseases is still being discussed^78^.

## Hypothalamic control of physical activity

Spontaneous physical activity (SPA) and its contribution to energy expenditure (i.e., NEAT) play a significant role in the modulation of energy homeostasis and body weight in humans and rodent models^79,80^. The duration and magnitude of NEAT are reported to be lower in obese individuals^81^, and those with higher SPA gain less weight^82–84^. Similar to feeding behaviors, SPA greatly affects energy homeostasis and is under the control of several brain areas and neuropeptides. The latter have been previously reviewed^85^. The most well-characterized mediators are orexin peptides^86^, which act on several hypothalamic regions to increase activity. First, the injection of orexin A into the rostral lateral hypothalamus nucleus (rLH) of rats induces running independently of feeding behavior^87^ and increases locomotor activity^88^. The effect was abolished with the preadministration of the γ-aminobutyric acid (GABA) receptor agonist muscimol^88^. The neuronal population in the rLH is speculated to be glutamic acid decarboxylase 65 (GAD65)-expressing neurons, as chemogenetic deactivation of these neurons decreases locomotion and blunts the effect of orexin receptor blockers^89^.

Within the hypothalamus, orexin is produced by neurons in the LH (orexin neurons). Transgenic mice bearing Cre expression in orexin neurons and stereotaxic injection of viruses enable the modulation of orexin neurons in the LH. Using these with a chemogenetic approach, Zink et al. demonstrated that acute activation of orexin neurons increases moving time without changing food intake behavior in male mice, especially during the light-on cycle. Similarly, the deactivation of LH orexin neurons in female mice decreases movement and energy expenditure, specifically in the dark cycle^90^. Long-term activation of orexin neurons in the LH also protects against diet-induced obesity through an increase in spontaneous activity and energy expenditure^90^. This finding is in accordance with the 24-h optogenetic activation of channelrhodopsin-2/orexin-Cre mice^80^.

It has been reported that the activation of LH GABAergic neurons using a chemogenetic approach induces a high level of locomotor activity, which is accompanied by an increase in energy expenditure^91^. Interestingly, another population of LH neurons—galanin-expressing neurons—that partially overlaps with the LH GABAergic neuron population can also induce an increase in locomotor activity when activated. However, galanin neurons reduce compulsive activity, while GABAergic neurons promote compulsive-like behaviors^91^.

The injection of orexin A into the PVN also increases locomotor activity, but the effect cannot be blocked by pretreatment with muscimol^88^. Another orexigenic neuropeptide that exerts an effect on physical activity through the PVN is neuropeptide Y (NPY). Hamsters in which NPY is injected into the PVN show increases in wheel running and food foraging behaviors^92^. NPY causes an increase in the intracellular level of Ca^2+^ and a decrease in the synaptic inputs of PVN neurons. The energy expenditure modulation effect of NPY in the PVN is due to the inhibition of endocannabinoid signaling^93^. However, the infusion of NPY into the PVN in rats cannot induce a change in locomotion^94^, and the overexpression of NPY in the PVN through a viral vector in rats cannot decrease the activity level^95^. This may be due to the spread of NPY when the PVN is infused with NPY instead of being locally injected^94^ or to the difference between the acute application versus the long-term expression of NPY in PVN neurons.

Other genes expressed in the PVN, such as BDNF^96^ or the BDNF TrkB receptor, have also been identified to be related to physical activity^97^. The knockout of either of these genes from PVN Sim1-expressing neurons causes a decrease in ambulatory activity in the dark phase. Interestingly, the deletion of the circadian clock-related transcription factor brain and muscle ARNT-like 1 (BMAL1) or GABA-A receptor γ2 subunit from PVN neurons decreases and disrupts the diurnal rhythm locomotion change^98^.

**Fig. 1:**
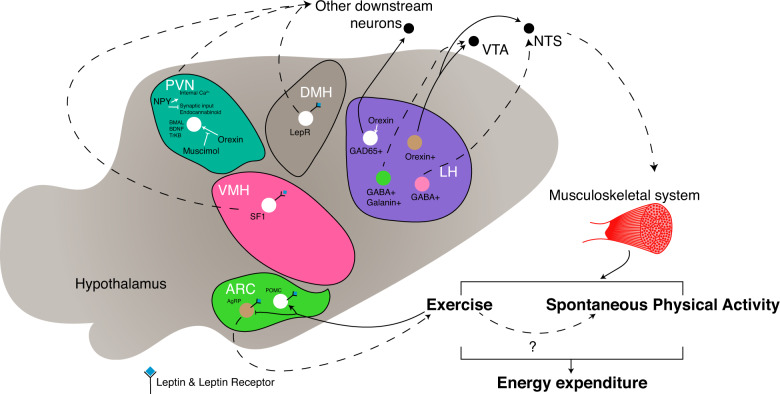
Schematic summary of the hypothalamic nuclei involved in the regulation of physical activity. Different neuronal populations and nuclei have different effectors and mechanisms to promote physical activities in the form of spontaneous physical activity or exercise, resulting in an increase in energy expenditure. Dashed lines: putative or unclear pathways or effects. Arrowheads: inducing or projecting. Blunted-bar heads: inhibiting. AgRP, agouti-related peptide; ARC, arcuate nucleus of the hypothalamus; BDNF, brain-derived neurotrophic factor; DMH, dorsomedial hypothalamus; GABA, gamma-aminobutyric acid; LepR, leptin receptor long form; LH, lateral hypothalamus; POMC, proopiomelanocortin; PVN, paraventricular hypothalamus; SF-1, steroidogenic factor 1; TrkB, tropomyosin receptor kinase B; VMH, ventromedial hypothalamus.

On the other hand, exercising is an intense metabolic challenge to the body, which requires adaptation and changes in energy homeostasis. Exercise has been shown to bring about neuronal and metabolic improvement, especially changes in the hormonal responses of the hypothalamic–pituitary–adrenal (HPA) axis^99–101^. In addition, hypothalamic neurons, specifically the POMC and agouti-related peptide (AgRP) neurons in the ARC, respond dynamically to exercise (e.g., a fast increase in the excitatory input and excitability of POMC neurons and the inhibition of NPY/AgRP neurons)^102^. This suggests that the hypothalamus plays a role in the control and feedback of physical activity. The hypothalamic control of physical activity is briefly summarized in Fig. 1.

Indeed, several lines of evidence have suggested that the hypothalamus (especially the ARC) contributes to the control of locomotor activity. First, the injection of ghrelin and agouti-related protein fragment (83–132), but not NPY, decreases the locomotor activity of rats over a period of 72 h^103^. In addition, the activation of a downstream target of leptin—STAT3—in AgRP neurons increases locomotor activity in mice^104^, while FoxO1 knockout in AgRP neurons decreases movement counts^54,105^. Furthermore, Rho-associated coiled-coil containing protein kinase 1 (ROCK1) knockout in either POMC or AgRP neurons causes a decrease in locomotion activity^106,107^. However, the mechanism behind the influence of AgRP neurons on locomotor activity is still unclear.

One explanation for the impact of AgRP neurons on ambulatory movement is through leptin receptor signaling. This explanation was supported by the evidence that the selective re-expression of leptin receptor in *Lepr*-null-allele-carrying mice causes an increase in beam break counts during calorimeter studies^108^, to a comparable extent as when leptin was injected into *ob/ob* mice^109^. This may be related to the ability of leptin to prevent the onset of torpor^110,111^. The role of leptin in torpor and thermogenesis is well discussed by Jan Nedergaard’s group^112^.

In addition to genetic regulation, epigenetic factors can contribute to the voluntary exercise behavior of mice. For example, DNA methylation of the AgRP neurons of *Dnmt3a* AgRP-specific-knockout mice causes a reduction in home cage activity and physical activity when the mice were presented with running wheels, without any changes in physical endurance^113^. The abolishment of *Dnmt3a* in AgRP neurons paradoxically increases CpG methylation in ARC neurons and changes the expression of melanocortin system-related genes, along with GABA transmitter genes in AgRP neurons^113^.

Another finding to support the notion that the ARC may control voluntary exercise is that AgRP neurons in the hypothalamus are quickly inhibited within 30 s at the end of a voluntary running session^102,114^, while POMC neurons are activated^102^. Using the activity-based anorexia (ABA) mouse model^115^, the impaired activation of AgRP neurons in response to a negative energy balance induced compulsive exercise, and this sustained exercise led to death^114^. Indeed, when AgRP neurons were ablated, female mice running under the ABA paradigm experienced white adipose tissue (WAT) shrinkage and brown adipose tissue atrophy, while the activation of this population increased the running counts and abilities of the food-restricted mice^114^. These findings provide one line of evidence for the role of AgRP neurons in compulsive exercise in individuals with anorexia nervosa.

In addition to the ARC, the VMH is also a central hub controlling the body’s response to exercise. The role of the VMH in orchestrating the sympathetic nervous system, fat metabolism and exercise benefits has been extensively reviewed^116^. Furthermore, lesions of the VMH cause hyperactivation^117^ and an increase in motivation for food in rats^118^. The global germline knockout of SF-1—an abundantly expressed and highly restricted nuclear receptor in the VMH—in female mice causes a significant long-term decrease in physical activity (in terms of wheel turns)^119^. This decline in voluntary exercise precedes the increase in body weight and is not observed in ovariectomized mice^119^. However, postnatal VMH-specific steroidogenic factor 1 (SF-1) knockout using CamKII-Cre does not lead to any difference in locomotor activity^120^. The role of SF-1 neurons in the regulation of physical activity is further compounded, as chemogenetic activation of SF-1 neurons using DREADD systems was reported to either not affect locomotor activity^121–123^ or decrease movement and rearing activities^124^. Furthermore, the inhibition of SF-1 neurons using the DREADD system can lead to an increase in spontaneous exploratory motions^124^ or not^125^. This discrepancy might be due to differences in experimental paradigms (for example, viral delivery of DREADD receptors versus a transgenic mouse line, different ligands used to activate DREADD receptor hM3Dq, different ligand concentrations and methods of delivery, and different parameters reported for “locomotor activity”). Further studies need to be performed to fully understand the impact of SF-1 neurons on physical activity.

Another neuronal population of the VMH is reported to increase locomotor activity, rearing movement, and heat generation in female mice when activated. This subset of neurons is estrogen-responsive and NK2 homeobox 1 (Nkx2-1)-positive and is located in the ventrolateral region of the VMH. This physical activity modulation requires estrogen signaling^123^. In mice with Nkx2-1 deletion using SF-1-Cre, physical activity is significantly less than that of wild-type mice, with less movement and lower cycling ability^123^.

## Hypothalamic control of diet-induced thermogenesis

### The arcuate nucleus of the hypothalamus

Over the past decades, it has become clear that hypothalamic nuclei, especially the arcuate nucleus of the hypothalamus (ARC), play an important role in the regulation of feeding and metabolism^126,127^. The ARC is located near the floor of the third ventricle and the median eminence with its fenestrated epithelium; hence, this area can sense nutritional status. The ARC is a key site for coordinated feedback responses to metabolic signals from hormones such as insulin and leptin, nutrients such as glucose and free fatty acids^128–130^, and neuronal inputs from other hypothalamic areas and extrahypothalamic areas^131^. The ARC includes anorexic POMC neurons and orexigenic NPY/AgRP neurons that have opposite effects on the regulation of energy homeostasis. These neurons have abundant projections into several brain areas that regulate the neuroendocrine system and metabolism. Recently, researchers have suggested a possible contribution from the ARC and its projections to secondary regions to the regulation of the production of BAT-related genes and thermogenesis^132^.

The most characterized pathway of the ARC for the regulation of energy homeostasis is through the melanocortin system. The brain melanocortin system is a critical neural system for the maintenance of body weight and energy expenditure^133,134^. The “first-order” neurons of the central melanocortin system constitute neurons that express the precursor POMC, which will be cleaved to generate alpha-melanocyte-stimulating hormone (α-MSH), and neurons that produce AgRP, an endogenous inverse agonist of melanocortin receptors^135^. Under satiated conditions, POMC neurons induce thermogenesis in BAT mainly through α-MSH-mediated activation of melanocortin 3 and 4 receptors (MC3/4R)^136^. The relationship between the melanocortin system and BAT thermogenesis has been discussed in several reviews^137^. Recently, a number of genetic factors have been identified to be crucial for adaptive postprandial thermogenesis in POMC neurons. First, POMC-specific transcriptional coactivator peroxisome proliferator-activated receptor γ (PPARγ) coactivator-1β (PGC-1β) deletion leads to higher body temperature during fed states or after being refed following 24 h of fasting^138^. This effect is due to an increase in leptin-induced thermogenesis sensitivity^138^. On the other hand, the disruption of the unfolded protein response and endoplasmic reticulum (ER) stress through interference with the inositol-requiring enzyme 1/X-box binding protein 1 (Ire1-Xbp1) pathway causes an inability to induce thermogenesis on a high-fat diet, thus causing obesity^139^. The ablation of Ire1α from POMC neurons renders these neurons leptin- and insulin-insensitive under endoplasmic reticulum stress^139^.

In contrast, the activation of NPY-expressing neurons in the ARC has been shown to inhibit sympathetic activation of BAT through direct Y1 receptor-mediated signal regulation in the paraventricular nucleus of the hypothalamus (PVN)^140^. Evidence in a recent report shows that the inactivation of AgRP neurons induces the browning of WAT and prevents diet-induced obesity and insulin resistance in mice, demonstrating the importance of AgRP neurons in body temperature regulation^141^.

Hormonal signals such as leptin and insulin in the ARC can regulate BAT thermogenesis^142^. There is evidence that leptin signaling plays a pivotal role in mediating sympathetic nervous system (SNS) tones in the ARC^143^. The homeostatic hormone leptin acts on the ARC through its receptor, inducing POMC expression, which increases the release of POMC products to secondary neurons. In the ARC, leptin also activates RIP-Cre-expressing neurons, which act on the PVN and generate heat through the inhibition of the GABAergic population^144^. Furthermore, the combined action of leptin in POMC neurons increases UCP1 expression and temperature in BAT by inhibiting tyrosine phosphatase 1B (PTP1B) and T-cell protein tyrosine phosphatase (TCPTP) signaling^144^. In addition to its role in carbohydrate metabolism, insulin is associated with feeding-related thermogenesis^145^. Mice fed a cafeteria diet exhibit spontaneous hyperphagia, increased energy expenditure, decreased insulin resistance, and a thermogenic response to noradrenaline and BAT enlargement^146^. However, in streptozotocin-diabetic rats, cafeteria feeding did not increase the response to resting VO_2_ or noradrenaline, and intraperitoneal injection of protamine zinc insulin after cafeteria-diet feeding leads to impaired thermogenesis in diabetic mice^145^. These findings suggest that cafeteria-diet-induced thermogenesis is insulin-dependent.

Recent studies in rodents show that NPY neurons located in the arcuate nucleus promote diet-induced heat generation through the neuropeptide FF receptor 2 (NPFFR2) signaling pathway. A deficiency of NPFFR2 results in decreased levels of UCP-1 and PGC-1α in BAT and, consequently, decreased thermogenesis in BAT. Together, these data provide evidence for an arcuate nucleus NPY-dependent circuit to control the thermogenesis of BAT^147^.

### The ventromedial hypothalamus

The ventromedial hypothalamus (VMH), spanning across the mediobasal hypothalamic area^148^, is a bilateral egg-shaped nucleus whose roles in controlling tissue thermogenesis were defined early. Early researchers demonstrated the control of the VMH over brown adipose tissue through stereotaxic lesions and electrical stimulation experiments on the VMH: some VMH lesion studies did not show any change in food intake, whereas some studies showed that the disruption of this region results in an increase in body weight and obesity^149^, a decrease in core body temperature^150^ and a decrease in postprandial brown adipose tissue sympathetic activation^151^. Some researchers have shown that adult pair-tube-fed rats with VMH destructions^152,153^ or rat weanlings with VMH lesions^154,155^ increase body weight without any difference in food consumption. These results demonstrated that the body weight gain from VMH lesions is due to not only the increase in food intake but also probably a disturbance in metabolism and thermogenesis^156^, which is supported by the change in the basal insulin level^157^ and fat disposition. Indeed, when electrical stimulation occurs through stereotaxically implanted electrodes in the VMH, a biphasic response in interscapular BAT temperature is observed, with an initial decrease of ~0.16 °C after 1 min, and an increase of almost 1 °C in ~10 min and a return to the initial value after ~20 min^158^. The rise in BAT temperature is followed by no or a minor change in quadriceps (skeletal) temperature. This response mimics the result of direct sympathetic nerve stimulation^159^ and can be triggered by an intraperitoneal injection of noradrenaline. Furthermore, the rise in BAT temperature is abolished by blocking β-adrenergic signaling using propranolol or by BAT nerve blockade through the injection of tetracaine into the interscapular area^158^, further suggesting that the VMH is involved in BAT thermogenesis through sympathetic outflow and innervation.

As a satiety signal, leptin also acts on the VMH to control feeding-related thermogenesis. Direct infusion of leptin within the VMH increases sympathetic-nerve-dependent glucose uptake of BAT^160^ and sympathetic nervous system tone, characterized by an increase in blood pressure, renal sympathetic outflow, and plasma catecholamine levels^161,162^. These data provide evidence for leptin action on the VMH to control heat generation from BAT. The role of the sympathetic nervous system in the regulation of thermogenesis will be covered by another review in this collection.

Highly populated in the VMH, SF-1 neurons can be one of the markers for this region^148,163^^,^^164^. The development of Cre recombinase-expressing mice under the driver of SF-1 enabled the genetic modulation and study of the role of the VMH in thermogenesis^163,165,166^. Interestingly, a major population of SF-1 neurons expresses leptin receptor^148^, and SF-1 Cre mouse lines were used to delete leptin receptor from the VMH. Mice lacking leptin receptor in SF-1 neurons are obese, with increased fat composition and decreased energy expenditure, even when food intake is normal on a standard chow diet^166,167^. When given a high-fat diet, leptin receptor knockout from SF-1 neurons causes susceptibility to diet-induced obesity and aberrant adaptive thermogenesis, with a gradual difference in caloric intake^166,167^.

One of the downstream factors in leptin signaling in the hypothalamus is the transcription factor FoxO1^168–170^. Through the knockout of FoxO1 in SF-1 neurons in the VMH, Kim et al. observed a lean phenotype^171^. FoxO1 knockout in SF-1 neurons protected mice from high-calorie-induced obesity, with higher energy expenditure before and during a high-fat challenge and increased levels of serum catecholamines and UCP1 expression in brown adipose tissue. These mice also displayed higher leptin and insulin sensitivity^171^. Interestingly, the disruption of SF-1 neurons by the postnatal deletion of SF-1 using CamKII-Cre also impairs energy expenditure with a high-fat diet, decreases UCP1 expression in the brown adipose tissue, and blunts leptin action^120^. Collectively, these results suggested that leptin signaling in SF-1 neurons in the VMH might modulate sympathetic nervous system and brown adipose tissue heat generation to protect against diet-induced obesity.

Furthermore, the energy gauge AMPK has gained attention as a converging mediator of adaptive thermogenesis. VMH AMPK relays the effect of different hormones (for example, insulin, leptin, and thyroidal hormones) and drugs (nicotine and liraglutide) to modulate sympathetic outflow^172^. AMPK subunit α1, but not α2, is speculated to be the negative regulator of thermogenesis, as the overexpression of the dominant-negative form of AMPKα1, in rats and mice prevents diet-induced obesity by increasing heat generation and brown fat function^173^. In addition, the ablation of AMPK α1 in SF-1 neurons in mice leads to an increase in brown fat temperature, brown fat sympathetic nerve activity, UCP1, and thermogenic genes in brown and white adipose tissues in a feeding-independent manner^173^. Several lines of evidence have demonstrated that VMH AMPK may be involved in the regulation of sympathetic outflow to BAT through the AMPK/acetyl-CoA carboxylase (ACC)/carnitine palmitoyltransferase 1 C (CPT1C) pathway. First, the deletion of AMPK α1 is accompanied by a decrease in the phosphorylation of hypothalamic pACCα^173^. Second, CPT1C knockout blunts the thermogenic effects of a high-fat diet or leptin administration, and the rescue of CPT1C in the VMH is sufficient to restore the proper diet-induced response of body temperature^174^. AMPK in the VMH is also suggested to be involved in the beiging of WAT. Hormonal signals, such as thyroid hormones^175,176^, estradiol^49,177^, leptin^178^, and GLP1 analogs^179^, converge in the VMH and exert their metabolic actions through the modulation of AMPK and the sympathetic nervous system. Taken together, these observations show that AMPK is an important key in the VMH–sympathetic nervous system–adipose tissues axis to control thermogenesis in BAT and the browning of WAT to shift the energy balance.

### Preoptic area–dorsomedial hypothalamus leptin signaling

The preoptic area (POA) is a large region lying in the rostral part of the hypothalamus and comprises the median and ventrolateral preoptic nuclei (MnPO and VLPO, respectively), the medial and lateral preoptic areas, and the suprachiasmatic nucleus^180^. Neurons in the POA project to the dorsomedial nucleus of the hypothalamus (DMH), and the projections are known to regulate body thermogenesis based on ambient temperatures (discussed later). Interestingly, both POA and DMH neurons express the long form of leptin receptor^181^, suggesting that these regions might be involved in the thermogenic effect of food intake and leptin. Many pieces of evidence support the thermoregulatory roles of leptin receptor-expressing neurons in the POA/DMH. First, leptin induces cFos expression in POA/DMH neurons^181–183^ and direct membrane potential responses in POA^184^ and DMH neurons under diet-induced obesity^183^. In addition, leptin-receptor-expressing neurons (LepR neurons) in the POA project to the DMH, and leptin-receptor-expressing neurons in both the POA and the DMH project to the medullary rostral raphe pallidus^181^. POA/DMH LepR neurons also connect to BAT^181^ and control BAT thermogenesis. The chemogenetic activation of glutamatergic LepR neurons in the POA decreases energy expenditure^185^, but leptin treatment in the POA does not change responses to ambient temperature changes. LepR neurons in the DMH increase energy expenditure^182^, and the ablation of LepR neurons in the DMH decreases heat generation and shifts metabolism toward the usage of fat^186^. Interestingly, POA LepR neurons are involved in the regulation of body temperature in response to nutritional status. Leptin signaling through leptin receptor in the POA is required for the reduction in the metabolism rate during fasting and is possibly involved in the regulation of energy expenditure through thyroid hormones when mice are on a high-fat diet^184^. Collectively, leptin signaling in the POA/DMH is important for thermogenesis adapting to feeding and energy states.

DMH NPY signaling is also reported to be involved in the browning of WAT. The knockdown of NPY in the DMH using shRNA-encoding adeno-associated virus (AAV) causes an increase in UCP1 expression in inguinal WAT through an increase in sympathetic nervous system input into WAT, thus ‘browning’ WAT^187^. However, it is unknown whether NPY-expressing neurons in the DMH are involved in the conventional POA/DMH–SNS–adipose tissue axis to regulate the sympathetic input of adipose tissues^187^.

## Hypothalamic control of cold-induced thermogenesis

To preserve body functions and homeostasis upon exposure to a cold environment, efferent pathways for heat production are activated. Different regions in the hypothalamus are responsible for cold-induced thermogenesis, both through shivering and nonshivering heat production.

The most prominent and well-established circuit that controls body temperature is the preoptic area (POA)–dorsomedial nucleus of the hypothalamus (DMH) circuit. The role of the POA–DMH circuit in the central regulation of responses to temperature change has been extensively reviewed^188,189^. In brief, the POA is known to receive sensory input from temperature-sensitive neurons. The thermogenic response upon cooling requires the activation of neurons in the MnPO and GABAergic inhibitory signals to the MPO^190^ or glutamatergic input to the DMH^191^. Blocking MnPO neuron activation with the GABA agonist muscimol ablates both shiver and nonshivering thermogenic responses to cold exposure^192^. Similarly, the inactivation of GABAergic neurons in the VLPO can elicit a hyperthermic response^193^. GABAergic VLPO neurons project to GABAergic and glutamatergic neurons in the DMH, whose activation induces quick rises in body temperature, energy, and physical activity^191,193^.

Furthermore, cold exposure induces Fos expression in the POA and DMH^194–197^. The Fos-immunoreactive cell distributions in the POA are different between cold- and hot-stimulus-receiving animals^197^, suggesting the segregation of cold-sensitive neurons. Neurons in the DMH are also reported to be activated by cold exposure. Cooling activates both GABAergic and glutamatergic neurons in the DMH, and the effect may come from sensory temperature signals rather than body temperature changes^193^. Direct cooling of the POA induces shivering responses^198^, while the warming of the POA with thermoprobes ablates this response^199,200^. This evidence suggests the existence of thermosensitive neurons in the hypothalamus that primarily respond to temperature changes. However, the characteristics of cold-responsive neurons (either directly or through an afferent from peripheral signals) are still rather ambiguous. Cold exposure induces action potentials and Ca^2+^ influx in primary cultured POA neurons, and this firing does not depend on the cold/menthol receptor TRPM8^201^. Recently, Viktor V. Feketa et al. identified cyclic nucleotide-gated cation channel 3 (CNGA3) in mice as a putative marker for POA cold-responsive neurons by comparing the Ca^2+^ influx and transcriptional maps between the dissociated POA neurons of mice and those of hibernating squirrels^202^. Using electrophysical approaches, the authors demonstrated that CNGA3 homomers and heteromers with CNGA1 induce currents in response to a decrease in temperature, and this effect is dependent on cyclic GMP^202^.

The VMH is another important nucleus involved in cold-induced thermogenesis, as the activation of the VMH induces BAT thermogenesis in cold-exposed rats^203^ and increases oxygen consumption and shivering in rabbits^204^, and chemical lesioning of the VMH causes cold intolerance in rats^205^. Takayuki Ishiwata and colleagues proposed that the VMH is not involved in thermogenesis under cold conditions, with evidence that extracellular noradrenaline, serotonin, and 3,4-dihydroxyphenylacetic acid (DOPAC) levels in the VMH are not changed during cold exposure^206^. However, the data showed that body temperature increase and sympathetic output were reflected through heart rate, while the cold challenge was blocked with the perfusion of tetrodotoxin into the VMH^206^. In addition, it is debatable whether microdialysis at the level of the lateral VMH can fully demonstrate neurotransmitter release from VMH neurons to downstream target regions, as the VMH does not directly innervate BAT^207^. In addition, genetic approaches have identified several genetic factors that are involved in thermogenesis during a cold stimulus. The role of the endocannabinoid system in SF-1 neurons was elaborated by knocking out monoacylglycerol hydrolase α/β-hydrolase domain 6 (ABHD6), which disturbs the endocannabinoid system and impairs thermogenic and cold-enduring ability^208^. Another key regulator, cyclin-dependent kinase 4 (CDK4), also showed importance in the cold-induced adaptive thermogenesis of SF-1 neurons, as the deletion of CDK4 increased cold resistance in mice, with increased sympathetic outflow and UCP1 expression in BAT^209^.

Maintaining body temperature during cold exposure also requires the action of thyroid hormones and regulation through the hypothalamus–pituitary–thyroid axis. The role of thyroid hormones and TRH in cold environments was reviewed^210^. This further highlights the role of the hypothalamus—especially the PVN—in the regulation of adaptive thermogenesis during cold exposure.

**Fig. 2:**
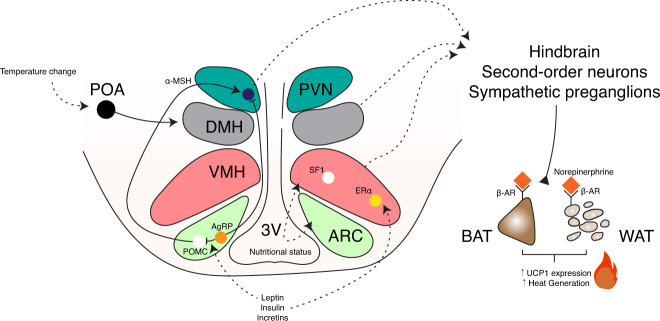
Schematic summary of the hypothalamic nuclei involved in the regulation of thermogenesis. Adaptive thermogenesis from nutritional or hormonal cues or from ambient temperature changes is controlled through different hypothalamic pathways. UCP1-dependent thermogenesis causes an increase in UCP1 expression in brown adipose tissue and “beiging” in white adipose tissue, thus increasing heat production. Arrowheads: inducing or projecting, Blunted-bar heads: inhibiting. 3V, third ventricle; αMSH, alpha-melanocyte-stimulating hormone; AgRP, agouti-related peptide; ARC, arcuate nucleus of the hypothalamus; β-AR, beta-adrenergic receptor; BAT, brown adipose tissue; ERα, estrogen receptor alpha; POMC, proopiomelanocortin; PVN, paraventricular hypothalamus; SF-1, steroidogenic factor; VMH, ventromedial hypothalamus; WAT, white adipose tissue.

Neurons in the PVN are also responsible for the synthesis of the hormone oxytocin. The disruption of oxytocin signaling through global knockout of oxytocin or oxytocin causes impairments in heat generation during the cold challenge period and lowers Fos-immunoreactive neurons in the DMH during cold challenges^211–213^. The re-expression of oxytocin receptor in the DMH and VMH in mice with global oxytocin receptor knockout suffices to reverse the cold intolerance phenotypes^213^, providing evidence of crosstalk between hypothalamic areas to control cold-induced thermogenesis. The relationship between hypothalamic nuclei and some adaptive thermogenesis mechanisms is depicted in Fig. 2.

Even though the input from the hypothalamus to browning adipose tissues has been well-established in rodent models, Rachid et al. suggested that there is no correlation between hypothalamus activity assessed by functional magnetic resonance imaging (fMRI) and the browning of adipose tissues or thermogenic gene expression in BAT^214^. However, the data suggested that in obese patients undergoing weight reduction, there is a blunted (and suggestively damaged) hypothalamus response after cold exposure^214^. This finding leads to questions regarding the translation of animal study findings to humans in clinical settings.

## Conclusion

Thermogenesis is a crucial component in the maintenance of energy homeostasis. Progress has been made in the understanding of the regulation of energy expenditure and thermogenesis, especially the central and hypothalamic control of this vital process. However, circuits and genetic factors involved in thermoregulation that we have not discovered may still exist. Further work is required to fully understand these metabolic pathways and to translate the findings into clinical contexts.

## References

[1] Morton GJ (2007). Hypothalamic leptin regulation of energy homeostasis and glucose metabolism. J. Physiol..

[2] Kim B (2008). Thyroid hormone as a determinant of energy expenditure and the basal metabolic rate. Thyroid.

[3] Saito M, Matsushita M, Yoneshiro T, Okamatsu-Ogura Y (2020). Brown adipose tissue, diet-induced thermogenesis, and thermogenic food ingredients: from mice to men. Front. Endocrinol..

[4] Tseng YH, Cypess AM, Kahn CR (2010). Cellular bioenergetics as a target for obesity therapy. Nat. Rev. Drug Discov..

[5] Dulloo AG, Seydoux J, Jacquet J (2004). Adaptive thermogenesis and uncoupling proteins: a reappraisal of their roles in fat metabolism and energy balance. Physiol. Behav..

[6] Campbell WW, Crim MC, Young VR, Evans WJ (1994). Increased energy requirements and changes in body-composition with resistance training in older adults. Am. J. Clin. Nutr..

[7] Johnstone AM, Murison SD, Duncan JS, Rance KA, Speakman JR (2005). Factors influencing variation in basal metabolic rate include fat-free mass, fat mass, age, and circulating thyroxine but not sex, circulating leptin, or triiodothyronine. Am. J. Clin. Nutr..

[8] Zurlo F, Nemeth PM, Choksi RM, Sesodia S, Ravussin E (1994). Whole-body energy-metabolism and skeletal-muscle biochemical characteristics. Metabolism.

[9] Zurlo F, Larson K, Bogardus C, Ravussin E (1990). Skeletal-muscle metabolism is a major determinant of resting energy-expenditure. J. Clin. Invest..

[10] Harris JA, Benedict FG (1918). A biometric study of human basal metabolism. Proc. Natl Acad. Sci. USA.

[11] Fleisch A (1951). Basal metabolism standard and its determination with the “metabocalculator”. Helv. Med. Acta.

[12] Hulbert AJ, Else PL (2004). Basal metabolic rate: history, composition, regulation, and usefulness. Physiol. Biochem. Zool..

[13] Ho KK (2018). Diet-induced thermogenesis: fake friend or foe?. J. Endocrinol..

[14] Margaria R, Edwards HT, Dill DB (1933). The possible mechanisms of contracting and paying the oxygen debt and the role of lactic acid in muscular contraction. Am. J. Physiol..

[15] Johannsen DL, Ravussin E (2008). Spontaneous physical activity: relationship between fidgeting and body weight control. Curr. Opin. Endocrinol. Diabetes Obes..

[16] Leitner BP (2017). Mapping of human brown adipose tissue in lean and obese young men. Proc. Natl Acad. Sci. USA.

[17] Herz CT, Kiefer FW (2019). Adipose tissue browning in mice and humans. J. Endocrinol..

[18] Ikeda K, Yamada T (2020). UCP1 Dependent and independent thermogenesis in brown and beige adipocytes. Front. Endocrinol..

[19] Bligh J, Johnson KG (1973). Glossary of terms for thermal physiology. J. Appl. Physiol..

[20] van Marken Lichtenbelt W (2012). Brown adipose tissue and the regulation of nonshivering thermogenesis. Curr. Opin. Clin. Nutr. Metab. Care.

[21] Nakamura K (2011). Central circuitries for body temperature regulation and fever. Am. J. Physiol. Regul. Integr. Comp. Physiol..

[22] Rolfe DF, Brown GC (1997). Cellular energy utilization and molecular origin of standard metabolic rate in mammals. Physiol. Rev..

[23] Abreu-Vieira G, Xiao C, Gavrilova O, Reitman ML (2015). Integration of body temperature into the analysis of energy expenditure in the mouse. Mol. Metab..

[24] Clarke MV, Russell PK, Zajac JD, Davey RA (2019). The androgen receptor in the hypothalamus positively regulates hind-limb muscle mass and voluntary physical activity in adult male mice. J. Steroid Biochem. Mol. Biol..

[25] Fan W (2005). Androgen receptor null male mice develop late-onset obesity caused by decreased energy expenditure and lipolytic activity but show normal insulin sensitivity with high adiponectin secretion. Diabetes.

[26] Wang Z, Khor S, Cai D (2020). Regulation of muscle and metabolic physiology by hypothalamic erythropoietin independently of its peripheral action. Mol. Metab..

[27] Kaiyala KJ (2010). Identification of body fat mass as a major determinant of metabolic rate in mice. Diabetes.

[28] Hyland L, Park SB, Abdelaziz Y, Abizaid A (2020). Ghrelin infused into the dorsomedial hypothalamus of male mice increases food intake and adiposity. Physiol. Behav..

[29] Chen M (2019). G(s)alpha deficiency in the dorsomedial hypothalamus leads to obesity, hyperphagia, and reduced thermogenesis associated with impaired leptin signaling. Mol. Metab..

[30] Burke LK (2016). Sex difference in physical activity, energy expenditure and obesity driven by a subpopulation of hypothalamic POMC neurons. Mol. Metab..

[31] David A (2011). Evidence for a continuum of genetic, phenotypic, and biochemical abnormalities in children with growth hormone insensitivity. Endocr. Rev..

[32] Velloso CP (2008). Regulation of muscle mass by growth hormone and IGF-I. Br. J. Pharm..

[33] Collett-Solberg PF (2019). Diagnosis, genetics, and therapy of short stature in children: a growth hormone research society international perspective. Horm. Res. Paediatr..

[34] Roth C (1997). Short stature and failure of pubertal development in thalassaemia major: evidence for hypothalamic neurosecretory dysfunction of growth hormone secretion and defective pituitary gonadotropin secretion. Eur. J. Pediatr..

[35] Cheung LYM, Okano H, Camper SA (2017). Sox21 deletion in mice causes postnatal growth deficiency without physiological disruption of hypothalamic-pituitary endocrine axes. Mol. Cell. Endocrinol..

[36] Lee B (2018). Dlx1/2 and Otp coordinate the production of hypothalamic GHRH- and AgRP-neurons. Nat. Commun..

[37] Lee J (2020). Insulin synthesized in the paraventricular nucleus of the hypothalamus regulates pituitary growth hormone production. JCI Insight.

[38] Henry CJ (2000). Mechanisms of changes in basal metabolism during ageing. Eur. J. Clin. Nutr..

[39] Fukagawa NK, Bandini LG, Young JB (1990). Effect of age on body composition and resting metabolic rate. Am. J. Physiol..

[40] Elia M, Ritz P, Stubbs RJ (2000). Total energy expenditure in the elderly. Eur. J. Clin. Nutr..

[41] Jiang CH, Tsien JZ, Schultz PG, Hu Y (2001). The effects of aging on gene expression in the hypothalamus and cortex of mice. Proc. Natl Acad. Sci. USA.

[42] Kim K, Choe HK (2019). Role of hypothalamus in aging and its underlying cellular mechanisms. Mech. Ageing Dev..

[43] Liu, T., Xu, Y., Yi, C. X., Tong, Q. & Cai, D. The hypothalamus for whole-body physiology: from metabolism to aging. *Protein Cell*10.1007/s13238-021-00834-x (2021). 10.1007/s13238-021-00834-x PMC9095790 33826123

[44] Yang SB (2012). Rapamycin ameliorates age-dependent obesity associated with increased mTOR signaling in hypothalamic POMC neurons. Neuron.

[45] Li G, Zhang Y, Wilsey JT, Scarpace PJ (2005). Hypothalamic pro-opiomelanocortin gene delivery ameliorates obesity and glucose intolerance in aged rats. Diabetologia.

[46] Yaswen L, Diehl N, Brennan MB, Hochgeschwender U (1999). Obesity in the mouse model of pro-opiomelanocortin deficiency responds to peripheral melanocortin. Nat. Med..

[47] McMurphy T (2019). Hypothalamic gene transfer of BDNF promotes healthy aging in mice. Aging Cell.

[48] Tumer N (2006). Hypothalamic rAAV-mediated GDNF gene delivery ameliorates age-related obesity. Neurobiol. Aging.

[49] Martinez de Morentin PB (2014). Estradiol regulates brown adipose tissue thermogenesis via hypothalamic AMPK. Cell Metab..

[50] Saito K (2016). PI3K in the ventromedial hypothalamic nucleus mediates estrogenic actions on energy expenditure in female mice. Sci. Rep..

[51] van Veen JE (2020). Hypothalamic estrogen receptor alpha establishes a sexually dimorphic regulatory node of energy expenditure. Nat. Metab..

[52] Malyala A, Zhang C, Bryant DN, Kelly MJ, Ronneklev OK (2008). PI3K signaling effects in hypothalamic neurons mediated by estrogen. J. Comp. Neurol..

[53] Park CJ (2011). Genetic rescue of nonclassical ERalpha signaling normalizes energy balance in obese Eralpha-null mutant mice. J. Clin. Invest..

[54] Ren H (2012). FoxO1 target Gpr17 activates AgRP neurons to regulate food Intake. Cell.

[55] Reilly AM (2019). Gpr17 deficiency in POMC neurons ameliorates the metabolic derangements caused by long-term high-fat diet feeding. Nutr. Diabetes.

[56] Silva JE (2003). The thermogenic effect of thyroid hormone and its clinical implications. Ann. Intern. Med..

[57] Silva JE (1988). Full expression of uncoupling protein gene requires the concurrence of norepinephrine and triiodothyronine. Mol. Endocrinol..

[58] Merchenthaler I, Liposits Z (1994). Mapping of thyrotropin-releasing hormone (TRH) neuronal systems of rat forebrain projecting to the median eminence and the OVLT. Immunocytochemistry combined with retrograde labeling at the light and electron microscopic levels. Acta Biol. Hung..

[59] Lechan RM, Qi Y, Jackson IM, Mahdavi V (1994). Identification of thyroid hormone receptor isoforms in thyrotropin-releasing hormone neurons of the hypothalamic paraventricular nucleus. Endocrinology.

[60] Fekete C, Lechan RM (2007). Negative feedback regulation of hypophysiotropic thyrotropin-releasing hormone (TRH) synthesizing neurons: role of neuronal afferents and type 2 deiodinase. Front. Neuroendocrinol..

[61] Lopez M (2010). Hypothalamic AMPK and fatty acid metabolism mediate thyroid regulation of energy balance. Nat. Med..

[62] Silva JE (2006). Thermogenic mechanisms and their hormonal regulation. Physiol. Rev..

[63] Prevot V (2018). The versatile tanycyte: a hypothalamic integrator of reproduction and energy metabolism. Endocr. Rev..

[64] Fonseca TL (2013). Coordination of hypothalamic and pituitary T3 production regulates TSH expression. J. Clin. Invest..

[65] Sanchez E (2009). Tanycyte pyroglutamyl peptidase II contributes to regulation of the hypothalamic-pituitary-thyroid axis through glial-axonal associations in the median eminence. Endocrinology.

[66] Muller-Fielitz H (2017). Tanycytes control the hormonal output of the hypothalamic-pituitary-thyroid axis. Nat. Commun..

[67] Rodriguez-Rodriguez A (2019). Tanycytes and the control of thyrotropin-releasing hormone flux into portal capillaries. Front. Endocrinol..

[68] Frare C, Williams CT, Drew KL (2021). Thermoregulation in hibernating mammals: the role of the “thyroid hormones system”. Mol. Cell. Endocrinol..

[69] Herwig A, Ross AW, Nilaweera KN, Morgan PJ, Barrett P (2008). Hypothalamic thyroid hormone in energy balance regulation. Obes. Facts.

[70] Cannon B, Nedergaard J (2011). Nonshivering thermogenesis and its adequate measurement in metabolic studies. J. Exp. Biol..

[71] Maloney SK, Fuller A, Mitchell D, Gordon C, Overton JM (2014). Translating animal model research: does it matter that our rodents are cold?. Physiology.

[72] Gordon CJ (2012). Thermal physiology of laboratory mice: defining thermoneutrality. J. Therm. Biol..

[73] Nedergaard J, Cannon B (2014). The browning of white adipose tissue: some burning issues. Cell Metab..

[74] Scholander PF, Andersen KL, Krog J, Lorentzen FV, Steen J (1957). Critical temperature in Lapps. J. Appl. Physiol..

[75] Hill RW, Muhich TE, Humphries MM (2013). City-scale expansion of human thermoregulatory costs. PLoS ONE.

[76] Castillo M (2011). Disruption of thyroid hormone activation in type 2 deiodinase knockout mice causes obesity with glucose intolerance and liver steatosis only at thermoneutrality. Diabetes.

[77] Feldmann HM, Golozoubova V, Cannon B, Nedergaard J (2009). UCP1 ablation induces obesity and abolishes diet-induced thermogenesis in mice exempt from thermal stress by living at thermoneutrality. Cell Metab..

[78] Seeley RJ, MacDougald OA (2021). Mice as experimental models for human physiology: when several degrees in housing temperature matter. Nat. Metab..

[79] Kotz CM, Teske JA, Billington CJ (2008). Neuroregulation of nonexercise activity thermogenesis and obesity resistance. Am. J. Physiol. Regul. Integr. Comp. Physiol..

[80] Kotz CM, Perez-Leighton CE, Teske JA, Billington CJ (2017). Spontaneous physical activity defends against obesity. Curr. Obes. Rep..

[81] Levine James A, Vander Weg Mark W, Hill James O, Klesges Robert C (2006). Non-exercise activity thermogenesis: the crouching tiger hidden dragon of societal weight gain. Arterioscler. Thromb. Vasc. Biol..

[82] Shook RP (2015). Low levels of physical activity are associated with dysregulation of energy intake and fat mass gain over 1 year. Am. J. Clin. Nutr..

[83] Drenowatz C, Hill JO, Peters JC, Soriano-Maldonado A, Blair SN (2017). The association of change in physical activity and body weight in the regulation of total energy expenditure. Eur. J. Clin. Nutr..

[84] Teske JA, Billington CJ, Kuskowski MA, Kotz CM (2012). Spontaneous physical activity protects against fat mass gain. Int. J. Obes. (Lond.).

[85] Teske JA, Billington CJ, Kotz CM (2008). Neuropeptidergic mediators of spontaneous physical activity and non-exercise activity thermogenesis. Neuroendocrinology.

[86] Kotz CM (2006). Integration of feeding and spontaneous physical activity: role for orexin. Physiol. Behav..

[87] Kotz CM, Teske JA, Levine JA, Wang C (2002). Feeding and activity induced by orexin A in the lateral hypothalamus in rats. Regul. Pept..

[88] Kotz CM (2006). Orexin A mediation of time spent moving in rats: neural mechanisms. Neuroscience.

[89] Kosse C, Schöne C, Bracey E, Burdakov D (2017). Orexin-driven GAD65 network of the lateral hypothalamus sets physical activity in mice. Proc. Natl Acad. Sci. USA.

[90] Zink AN, Bunney PE, Holm AA, Billington CJ, Kotz CM (2018). Neuromodulation of orexin neurons reduces diet-induced adiposity. Int. J. Obes..

[91] Qualls-Creekmore E (2017). Galanin-expressing GABA neurons in the lateral hypothalamus modulate food reward and noncompulsive locomotion. J. Neurosci..

[92] Dailey MJ, Bartness TJ (2009). Appetitive and consummatory ingestive behaviors stimulated by PVH and perifornical area NPY injections. *Am*. J. Physiol. Regul. Integr. Comp. Physiol..

[93] Péterfi Z (2018). Endocannabinoid and nitric oxide systems of the hypothalamic paraventricular nucleus mediate effects of NPY on energy expenditure. Mol. Metab..

[94] van Dijk G, Strubbe JH (2003). Time-dependent effects of neuropeptide Y infusion in the paraventricular hypothalamus on ingestive and associated behaviors in rats. Physiol. Behav..

[95] Tiesjema B, la Fleur SE, Luijendijk MCM, Adan RAH (2009). Sustained NPY overexpression in the PVN results in obesity via temporarily increasing food intake. Obesity.

[96] An JJ (2015). Discrete BDNF neurons in the paraventricular hypothalamus control feeding and energy expenditure. Cell Metab..

[97] An JJ (2020). TrkB-expressing paraventricular hypothalamic neurons suppress appetite through multiple neurocircuits. Nat. Commun..

[98] Kim ER (2020). Paraventricular hypothalamus mediates diurnal rhythm of metabolism. Nat. Commun..

[99] Brenton TL (2016). Voluntary exercise improves hypothalamic and metabolic function in obese mice. J. Endocrinol..

[100] Anderson T, Berry NT, Wideman L (2019). Exercise and the hypothalamic–pituitary–adrenal axis: a special focus on acute cortisol and growth hormone responses. Curr. Opin. Endocr. Metab. Res..

[101] Uribe RM (2014). Voluntary exercise adapts the hypothalamus-pituitary-thyroid axis in male rats. Endocrinology.

[102] He Z (2018). Cellular and synaptic reorganization of arcuate NPY/AgRP and POMC neurons after exercise. Mol. Metab..

[103] Tang-Christensen M (2004). Central administration of ghrelin and agouti-related protein (83–132) increases food intake and decreases spontaneous locomotor activity in rats. Endocrinology.

[104] Mesaros A (2008). Activation of Stat3 signaling in AgRP neurons promotes locomotor activity. Cell Metab..

[105] Heinrich G, Meece K, Wardlaw SL, Accili D (2014). Preserved energy balance in mice lacking FoxO1 in neurons of Nkx2.1 lineage reveals functional heterogeneity of FoxO1 signaling within the hypothalamus. Diabetes.

[106] Huang H (2012). Rho-kinase regulates energy balance by targeting hypothalamic leptin receptor signaling. Nat. Neurosci..

[107] Huang H (2013). ROCK1 in AgRP neurons regulates energy expenditure and locomotor activity in male mice. Endocrinology.

[108] Coppari R (2005). The hypothalamic arcuate nucleus: a key site for mediating leptin’s effects on glucose homeostasis and locomotor activity. Cell Metab..

[109] Pelleymounter MA (1995). Effects of the obese gene product on body weight regulation in ob/ob mice. Science.

[110] Gavrilova O (1999). Torpor in mice is induced by both leptin-dependent and -independent mechanisms. Proc. Natl Acad. Sci. USA.

[111] Bolze F (2016). Long-acting PASylated leptin ameliorates obesity by promoting satiety and preventing hypometabolism in leptin-deficient Lep(ob/ob) mice. Endocrinology.

[112] Fischer AW, Cannon B, Nedergaard J (2020). Leptin: is it thermogenic?. Endocr. Rev..

[113] MacKay H (2019). DNA methylation in AgRP neurons regulates voluntary exercise behavior in mice. Nat. Commun..

[114] Miletta MC (2020). AgRP neurons control compulsive exercise and survival in an activity-based anorexia model. Nat. Metab..

[115] Routtenberg A, Kuznesof AW (1967). Self-starvation of rats living in activity wheels on a restricted feeding schedule. J. Comp. Physiol. Psychol..

[116] Fujikawa, T., Castorena, C. M., Lee, S. & Elmquist, J. K. The hypothalamic regulation of metabolic adaptations to exercise. *J. Neuroendocrinol*. **29**, 10.1111/jne.12533 (2017). 10.1111/jne.12533 PMC6264914 28887871

[117] Wiener NI, Nobrega J, Ossenkopp K-P, Shilman DM (1980). Acute hyperkinesia after hypothalamic lesions: a comparison of the time course, level, and type of hyperkinesia induced by ventromedial and lateral hypothalamic lesions in rats. Exp. Neurol..

[118] Wampler RS (1973). Increased motivation in rats with ventromedial hypothalamic lesions. J. Comp. Physiol. Psychol..

[119] Majdic G (2002). Knockout mice lacking steroidogenic factor 1 are a novel genetic model of hypothalamic obesity. Endocrinology.

[120] Kim KW (2011). Steroidogenic factor 1 directs programs regulating diet-induced thermogenesis and leptin action in the ventral medial hypothalamic nucleus. Proc. Natl Acad. Sci. USA.

[121] Coutinho EA (2017). Activation of SF1 neurons in the ventromedial hypothalamus by DREADD technology increases insulin sensitivity in peripheral tissues. Diabetes.

[122] Zhang J, Chen D, Sweeney P, Yang Y (2020). An excitatory ventromedial hypothalamus to paraventricular thalamus circuit that suppresses food intake. Nat. Commun..

[123] Correa SM (2015). An estrogen-responsive module in the ventromedial hypothalamus selectively drives sex-specific activity in females. Cell Rep..

[124] Viskaitis P (2017). Modulation of SF1 neuron activity coordinately regulates both feeding behavior and associated emotional states. Cell Rep..

[125] Silva BA (2016). The ventromedial hypothalamus mediates predator fear memory. Eur. J. Neurosci..

[126] Sainsbury A, Cooney GJ, Herzog H (2002). Hypothalamic regulation of energy homeostasis. Best. Pract. Res. Clin. Endocrinol. Metab..

[127] Timper K, Bruning JC (2017). Hypothalamic circuits regulating appetite and energy homeostasis: pathways to obesity. Dis. Model. Mech..

[128] Rodriguez EM, Blazquez JL, Guerra M (2010). The design of barriers in the hypothalamus allows the median eminence and the arcuate nucleus to enjoy private milieus: the former opens to the portal blood and the latter to the cerebrospinal fluid. Peptides.

[129] Cone RD (2001). The arcuate nucleus as a conduit for diverse signals relevant to energy homeostasis. Int. J. Obes. Relat. Metab. Disord..

[130] Myers MG, Olson DP (2012). Central nervous system control of metabolism. Nature.

[131] Wang D (2015). Whole-brain mapping of the direct inputs and axonal projections of POMC and AgRP neurons. Front. Neuroanat..

[132] Chitravanshi VC, Kawabe K, Sapru HN (2016). Stimulation of the hypothalamic arcuate nucleus increases brown adipose tissue nerve activity via hypothalamic paraventricular and dorsomedial nuclei. Am. J. Physiol. Heart Circ. Physiol..

[133] Cone RD (1999). The central melanocortin system and energy homeostasis. Trends Endocrinol. Metab..

[134] Gantz I, Fong TM (2003). The melanocortin system. Am. J. Physiol. Endocrinol. Metab..

[135] Ollmann MM (1997). Antagonism of central melanocortin receptors in vitro and in vivo by agouti-related protein. Science.

[136] Adan RAH (2006). The MC4 receptor and control of appetite. Br. J. Pharm..

[137] Labbé SM (2015). Hypothalamic control of brown adipose tissue thermogenesis. Front. Syst. Neurosci..

[138] Delezie J, Gill JF, Santos G, Karrer-Cardel B, Handschin C (2020). PGC-1β-expressing POMC neurons mediate the effect of leptin on thermoregulation in the mouse. Sci. Rep..

[139] Yao T (2017). Ire1α in Pomc neurons Is required for thermogenesis and glycemia. Diabetes.

[140] Shi YC (2013). Arcuate NPY controls sympathetic output and BAT function via a relay of tyrosine hydroxylase neurons in the PVN. Cell Metab..

[141] Ruan HB (2014). O-GlcNAc transferase enables AgRP neurons to suppress browning of white fat. Cell.

[142] Rahmouni K, Morgan DA (2007). Hypothalamic arcuate nucleus mediates the sympathetic and arterial pressure responses to leptin. Hypertension.

[143] Harlan SM (2011). Ablation of the leptin receptor in the hypothalamic arcuate nucleus abrogates leptin-induced sympathetic activation. Circ. Res..

[144] Kong D (2012). GABAergic RIP-Cre neurons in the arcuate nucleus selectively regulate energy expenditure. Cell.

[145] Rothwell NJ, Stock MJ (1981). A role for insulin in the diet-induced thermogenesis of cafeteria-fed rats. Metabolism.

[146] Sampey BP (2011). Cafeteria diet is a robust model of human metabolic syndrome with liver and adipose inflammation: comparison to high-fat diet. Obesity.

[147] Zhang L (2018). Diet-induced adaptive thermogenesis requires neuropeptide FF receptor-2 signalling. Nat. Commun..

[148] Choi Y-H, Fujikawa T, Lee J, Reuter A, Kim KW (2013). Revisiting the ventral medial nucleus of the hypothalamus: the roles of SF-1 neurons in energy homeostasis. Front. Neurosci..

[149] Gold RM (1973). Hypothalamic obesity: the myth of the ventromedial nucleus. Science.

[150] Monda M, Sullo A, De Luca V, Viggiano A, Pellicano MP (1997). Acute lesions of the ventromedial hypothalamus reduce sympathetic activation and thermogenic changes induced by PGE1. J. Physiol. Paris.

[151] Monda M, Sullo A, De Luca B (1997). Lesions of the ventromedial hypothalamus reduce postingestional thermogenesis. Physiol. Behav..

[152] Cox JE, Powley TL (1981). Intragastric pair feeding fails to prevent VMH obesity or hyperinsulinemia. Am. J. Physiol..

[153] Han PW (1968). Energy metabolism of tube-fed hypophysectomized rats bearing hypothalamic lesions. Am. J. Physiol..

[154] Bernardis LL, Frohman LA (1971). Effects of hyothalamic lesions at different loci on development of hyperinsulinemia and obesity in the weanling rat. J. Comp. Neurol..

[155] Frohman LA, Bernardis LL, Schnatz JD, Burek L (1969). Plasma insulin and triglyceride levels after hypothalamic lesions in weanling rats. Am. J. Physiol..

[156] Powley TL (1977). The ventromedial hypothalamic syndrome, satiety, and a cephalic phase hypothesis. Psychol. Rev..

[157] Berthoud HR, Jeanrenaud B (1979). Acute hyperinsulinemia and its reversal by vagotomy after lesions of the ventromedial hypothalamus in anesthetized rats. Endocrinology.

[158] Perkins MN, Rothwell NJ, Stock MJ, Stone TW (1981). Activation of brown adipose tissue thermogenesis by the ventromedial hypothalamus. Nature.

[159] Flaim KE, Horwitz BA, Horowitz JM (1977). Coupling of signals to brown fat: alpha- and beta-adrenergic responses in intact rats. Am. J. Physiol..

[160] Minokoshi Y, Haque MS, Shimazu T (1999). Microinjection of leptin into the ventromedial hypothalamus increases glucose uptake in peripheral tissues in rats. Diabetes.

[161] Marsh AJ (2003). Cardiovascular responses evoked by leptin acting on neurons in the ventromedial and dorsomedial hypothalamus. Hypertension.

[162] Montanaro MS, Allen AM, Oldfield BJ (2005). Structural and functional evidence supporting a role for leptin in central neural pathways influencing blood pressure in rats. Exp. Physiol..

[163] Sun JS (2021). Ventromedial hypothalamic primary cilia control energy and skeletal homeostasis. J. Clin. Invest..

[164] Yang DJ, Hong J, Kim KW (2021). Hypothalamic primary cilium: A hub for metabolic homeostasis. Exp. Mol. Med..

[165] Bingham NC, Verma-Kurvari S, Parada LF, Parker KL (2006). Development of a steroidogenic factor 1/Cre transgenic mouse line. Genesis.

[166] Dhillon H (2006). Leptin directly activates SF1 neurons in the VMH, and this action by leptin is required for normal body-weight homeostasis. Neuron.

[167] Bingham NC, Anderson KK, Reuter AL, Stallings NR, Parker KL (2008). Selective loss of leptin receptors in the ventromedial hypothalamic nucleus results in increased adiposity and a metabolic syndrome. Endocrinology.

[168] Kim M-S (2006). Role of hypothalamic Foxo1 in the regulation of food intake and energy homeostasis. Nat. Neurosci..

[169] Kitamura T (2006). Forkhead protein FoxO1 mediates Agrp-dependent effects of leptin on food intake. Nat. Med..

[170] Doan KV (2016). FoxO1 in dopaminergic neurons regulates energy homeostasis and targets tyrosine hydroxylase. Nat. Commun..

[171] Kim KW (2012). FOXO1 in the ventromedial hypothalamus regulates energy balance. J. Clin. Invest..

[172] Lage R, Ferno J, Nogueiras R, Dieguez C, Lopez M (2016). Contribution of adaptive thermogenesis to the hypothalamic regulation of energy balance. Biochem. J..

[173] Seoane-Collazo P (2018). SF1-specific AMPKα1 deletion protects against diet-induced obesity. Diabetes.

[174] Rodriguez-Rodriguez R (2019). CPT1C in the ventromedial nucleus of the hypothalamus is necessary for brown fat thermogenesis activation in obesity. Mol. Metab..

[175] Martínez-Sánchez N (2017). Hypothalamic AMPK-ER stress-JNK1 axis mediates the central actions of thyroid hormones on energy balance. Cell Metab..

[176] Martínez-Sánchez N (2017). Thyroid hormones induce browning of white fat. J. Endocrinol..

[177] Martínez de Morentin PB (2015). Pregnancy induces resistance to the anorectic effect of hypothalamic malonyl-CoA and the thermogenic effect of hypothalamic AMPK inhibition in female rats. Endocrinology.

[178] Tanida M, Yamamoto N, Shibamoto T, Rahmouni K (2013). Involvement of hypothalamic AMP-activated protein kinase in leptin-induced sympathetic nerve activation. PLoS ONE.

[179] Beiroa D (2014). GLP-1 Agonism stimulates brown adipose tissue thermogenesis and browning through hypothalamic AMPK. Diabetes.

[180] Saper CB, Lowell BB (2014). The hypothalamus. Curr. Biol..

[181] Zhang Y (2011). Leptin-receptor-expressing neurons in the dorsomedial hypothalamus and median preoptic area regulate sympathetic brown adipose tissue circuits. J. Neurosci..

[182] Rezai-Zadeh K (2014). Leptin receptor neurons in the dorsomedial hypothalamus are key regulators of energy expenditure and body weight, but not food intake. Mol. Metab..

[183] Lee SJ (2013). Leptin stimulates neuropeptide Y and cocaine amphetamine-regulated transcript coexpressing neuronal activity in the dorsomedial hypothalamus in diet-induced obese mice. J. Neurosci..

[184] Yu S (2018). Preoptic leptin signaling modulates energy balance independent of body temperature regulation. eLife.

[185] Yu S (2016). Glutamatergic preoptic area neurons that express leptin receptors drive temperature-dependent body weight homeostasis. J. Neurosci..

[186] Faber CL (2021). Leptin receptor neurons in the dorsomedial hypothalamus regulate diurnal patterns of feeding, locomotion, and metabolism. eLife.

[187] Chao P-T (2011). Knockdown of NPY expression in the dorsomedial hypothalamus promotes development of brown adipocytes and prevents diet-induced obesity. Cell Metab..

[188] Morrison SF, Nakamura K (2019). Central mechanisms for thermoregulation. Annu. Rev. Physiol..

[189] Tan CL, Knight ZA (2018). Regulation of body temperature by the nervous system. Neuron.

[190] Nakamura K, Morrison SF (2008). Preoptic mechanism for cold-defensive responses to skin cooling. J. Physiol..

[191] da Conceição EPS, Morrison SF, Cano G, Chiavetta P, Tupone D (2020). Median preoptic area neurons are required for the cooling and febrile activations of brown adipose tissue thermogenesis in rat. Sci. Rep..

[192] Nakamura K, Morrison SF (2011). Central efferent pathways for cold-defensive and febrile shivering. J. Physiol..

[193] Zhao ZD (2017). A hypothalamic circuit that controls body temperature. Proc. Natl Acad. Sci. USA.

[194] Yoshida K, Konishi M, Nagashima K, Saper CB, Kanosue K (2005). Fos activation in hypothalamic neurons during cold or warm exposure: Projections to periaqueductal gray matter. Neuroscience.

[195] Bratincsák A, Palkovits M (2004). Activation of brain areas in rat following warm and cold ambient exposure. Neuroscience.

[196] Bachtell RK, Tsivkovskaia NO, Ryabinin AE (2003). Identification of temperature-sensitive neural circuits in mice using c-Fos expression mapping. Brain Res.

[197] Uchida Y, Onishi K, Tokizawa K, Nagashima K (2018). Regional differences of cFos immunoreactive cells in the preoptic areas in hypothalamus associated with heat and cold responses in mice. Neurosci. Lett..

[198] Hammel HT, Hardy JD, Fusco MM (1960). Thermoregulatory responses to hypothalamic cooling in unanesthetized dogs. Am. J. Physiol..

[199] Hemingway A, Forgrave P, Birzis L (1954). Shivering suppression by hypothalamic stimulation. J. Neurophysiol..

[200] Kanosue K, Zhang YH, Yanase-Fujiwara M, Hosono T (1994). Hypothalamic network for thermoregulatory shivering. Am. J. Physiol..

[201] Abe J, Okazawa M, Adachi R, Matsumura K, Kobayashi S (2003). Primary cold-sensitive neurons in acutely dissociated cells of rat hypothalamus. Neurosci. Lett..

[202] Feketa VV, Nikolaev YA, Merriman DK, Bagriantsev SN, Gracheva EO (2020). CNGA3 acts as a cold sensor in hypothalamic neurons. eLife.

[203] Halvorson I, Gregor L, Thornhill JA (1990). Brown adipose tissue thermogenesis is activated by electrical and chemical (l-glutamate) stimulation of the ventromedial hypothalamic nucleus in cold-acclimated rats. Brain Res..

[204] Morimoto A, Murakami N, Ono T, Watanabe T, Sakata Y (1986). Stimulation of ventromedial hypothalamus induces cold defense responses in conscious rabbits. Am. J. Physiol..

[205] Preston E, Triandafillou J, Haas N (1989). Colchicine lesions of ventromedial hypothalamus: effects on regulatory thermogenesis in the rat. Pharmacol. Biochem. Behav..

[206] Ishiwata T, Greenwood BN (2018). Changes in thermoregulation and monoamine release in freely moving rats during cold exposure and inhibition of the ventromedial, dorsomedial, or posterior hypothalamus. J. Comp. Physiol. B.

[207] Cheung CC, Kurrasch DM, Liang JK, Ingraham HA (2013). Genetic labeling of steroidogenic factor-1 (SF-1) neurons in mice reveals ventromedial nucleus of the hypothalamus (VMH) circuitry beginning at neurogenesis and development of a separate non-SF-1 neuronal cluster in the ventrolateral VMH. J. Comp. Neurol..

[208] Fisette A (2016). α/β-Hydrolase domain 6 in the ventromedial hypothalamus controls energy metabolism flexibility. Cell Rep..

[209] Castillo-Armengol J (2020). Hypothalamic CDK4 regulates thermogenesis by modulating sympathetic innervation of adipose tissues. EMBO Rep..

[210] Zhang Z, Boelen A, Kalsbeek A, Fliers E (2018). TRH neurons and thyroid hormone coordinate the hypothalamic response to cold. Eur. Thyroid J..

[211] Kasahara Y, Takayanagi Y, Kawada T, Itoi K, Nishimori K (2007). Impaired thermoregulatory ability of oxytocin-deficient mice during cold-exposure. Biosci. Biotechnol. Biochem..

[212] Takayanagi Y (2008). Oxytocin receptor-deficient mice developed late-onset obesity. Neuroreport.

[213] Kasahara Y (2013). Oxytocin receptor in the hypothalamus is sufficient to rescue normal thermoregulatory function in male oxytocin receptor knockout mice. Endocrinology.

[214] Rachid B (2015). Distinct regulation of hypothalamic and brown/beige adipose tissue activities in human obesity. Int. J. Obes..

